# Seaweed extract improve drought tolerance of soybean by regulating stress-response genes

**DOI:** 10.1093/aobpla/plx051

**Published:** 2017-10-11

**Authors:** Pushp S Shukla, Katy Shotton, Erin Norman, Will Neily, Alan T Critchley, Balakrishnan Prithiviraj

**Affiliations:** Marine Bio-Products Research Laboratory, Department of Plant, Food and Environmental Sciences, Faculty of Agriculture, Dalhousie University, Truro, Nova Scotia, Canada; Research and Development, Acadian Seaplants Limited, Dartmouth, Nova Scotia, Canada

**Keywords:** *Ascophyllum nodosum*, commercial extract, drought stress, plant biostimulant, soybean, stomatal conductance

## Abstract

There is an increasing global concern about the availability of water for agricultural use. Drought stress negatively impacts plant physiology and crop productivity. Soybean (*Glycine max*) is one of the important oilseed crops, and its productivity is often reduced by drought. In this study, a commercial extract of *Ascophyllum nodosum* (ANE) was evaluated for its potential to alleviate drought stress in soybean. The aim of this study was to determine the effects of ANE on the response of soybean plants to drought stress by monitoring stomatal conductance, relative leaf water content, antioxidant activity and expression of stress-responsive genes. Plants treated with ANE had higher relative water content and higher stomatal conductance under drought stress. During early recovery in the post-drought phase, ANE treated plants had significantly higher stomatal conductance. The antioxidant activity was also found higher in the plants treated with ANE. In addition, ANE-treatment led to changes in the expression of stress-responsive genes: *GmCYP707A1a*, *GmCYP707A3b*, *GmRD22*, *GmRD20*, *GmDREB1B*, *GmERD1*, *GmNFYA3*, *FIB1a*, *GmPIP1b*, *GmGST*, *GmBIP* and *GmTp55*. Taken together, these results suggest that applications of ANE improve the drought tolerance of soybean by changing physiology and gene expression.

## Teaser

Ascophyllum nodosum extract (ANE) improved growth of soybean plants under drought stress. ANE treatment resulted in an increase in stomatal conductance and anti-oxidant activity in the leaf tissue. The application of ANE also modulated the expression of stress-responsive genes that ultimately resulted in improved growth under drought stress conditions. The results suggest ANE amplifies the plants' natural response to drought stress through change in the pattern of expression of stress response genes. This might be one plausible mode of action of ANE.

## Introduction

Abiotic stresses such as salinity, drought and temperature (heat and cold) have negative effects on plant productivity (Agarwal *et al.* 2013). Amongst these abiotic stresses, drought is one of the major factors limiting crop productivity in many parts of the world (Farooq *et al.* 2009). As a means of natural adaptation, plants have been equipped with a wide spectrum of physiological responses to mitigate the damaging effects of drought stress. The response of plants to such stressors is polygenic, complex and dynamic processes (Chaves *et al.* 2003). These adaptations include expression of stress-related genes, induction of biochemical responses, maintenance of root growth and water uptake, and reduced leaf area (Seki *et al.* 2007; Krasensky and Jonak 2012). It is well known that drought stress induces the accumulation of abscisic acid (ABA) which regulates stomatal closure thereby reducing photosynthetic activity (Chaves *et al.* 2009).

ABA-mediated stomatal closure is one of the first responses to drought stress (Hetherington and Woodward 2003). Although guard cells loose turgor as a result of a direct loss of water, stomatal closure, in response to dehydration, is always an active, energy-dependent process (Hetherington and Woodward 2003). Stomatal closure results in reduction of stomatal conductance and CO_2_ availability, which directly reduces rates of photosynthesis (Chaves *et al.* 2003). The response is accompanied by an increase in leaf temperature (Jones 1999). If leaf temperature reaches a threshold, it often leads to irreversible leaf tissue damage. Hence, leaf temperature can be used as an indicator of plant stress (Jones 1999; Jones *et al.* 2009).

Due to the complex metabolic pathways involved in drought tolerance, there has been limited success in generating drought-tolerant crop varieties by means of genetic engineering. Another approach to improve drought tolerance in plants is the use of plant biostimulants which are gaining major market acceptance (Khan *et al.* 2009; Jithesh *et al.* 2012). One category of product accepted as a biostimulant is seaweed extracts. Extracts of brown seaweeds are increasingly used in agricultural and horticultural crop production (Khan *et al.* 2009; du Jardin 2015; Battacharyya *et al.* 2015; Tandon and Dubey 2015). Soil or leaf applications of seaweed extract increase chlorophyll content, improve photosynthesis and nutrient uptake (Blunden *et al.* 1997), whilst also increasing water retention capacity and generally ameliorating biotic and abiotic stresses (Zhang and Schmidt 1999; Sangha *et al.* 2010; Subramanian *et al.* 2011). Studies have shown the positive effects of commercial extract of *Ascophyllum nodosum* on plants’ tolerance to stresses (Neily *et al.* 2010; Spann and Little 2011; Wally *et al.* 2013; Martynenko *et al.* 2016). However, the mode of action of seaweed extract in improving stress tolerance is not fully understood. Soybean (*Glycine max*) is one of the major oil-seed crops in the world and has a great economic and social value (Manavalan *et al.* 2009). Drought stress is perhaps the major constraint to the production and yield of this crop. In this study, we investigated the plausible mode of action of ANE in mitigating drought stress in soybean.

## Methods

### Plant growth and stress treatment

Soybean (*Glycine max*), variety Savana was planted in ProMix BX (Premier Tech Horticulture, Canada) in 200-seed trays, in a growth chamber with an 18/6 dark/light cycle with 400 µmol m^−2^ s spectral flux photons of photosynthetically active radiation (PAR) at 27 °C for 7 days. After germination, the plants were transplanted into 12 cm pots and placed in a Conviron® environmental chamber (Winnipeg, Canada) and grown for an additional 14 days, then the drought stress was initiated. The environmental chamber was set at 16:8 light/dark cycle, with 27 °C temperature, ~400 µmol m^−2^ s^−1^ PAR, 600 ppm CO_2_ and 60% relative humidity. Soybean plants were divided into two sets having 10 plants per set; twice weekly one set of the control plants were treated with a 100 mL solution of 0.5 g L^–1^ fertilizer per plant (20-8-20, Plant Products, Canada) and the other set, individual plants were treated with 0.5 g L^–1^ of the same fertilizer solution but with 7.0 mL L^–1^ ANE (Acadian®, Acadian Seaplants Limited, Dartmouth, Nova Scotia, Canada).Twenty-one days after planting, both the sets of plants were further treated with 1.0 g L^–1^ 20-8-20 and 1.0 g L^–1^ 20-8-20 plus 7.0 mL L^–1^ ANE, respectively, until the soil was completely saturated and excess liquid ran through the bottom of the pots (~300 mL). This equalized the soil moisture content for the onset of water stress. After this application, the plants were subjected to the drought stress by stopping irrigation. Measurements of different parameters and samples were taken at three time points related to three phases of stress, that is before stress (22 h after last treatment when the substrate was still wet), during stress (75 h after last treatment and recovery. In recovery phase, both the sets of plants were irrigated after 89 h after the last treatment and samples were collected 8 h after irrigation). For gene expression studies, the third set of trifoliate leaves were taken in triplicates from each treatment and directly frozen in liquid nitrogen to be stored at −80 °C.

### Determination of free-radical scavenging activity

Free-radical scavenging activity was evaluated, as described by Kähkönen *et al.* (1999); briefly a 200 mg leaf sample was homogenized in 15 mL of 80% methanol and centrifuged for 15 min at 5000 rpm at 4 °C. Two hundred and fifty microliters of the methanolic extracts from each sample were added to 250 μL methanol and 500 μL of 1, 1-diphenyl-2-picrylhydrazyl (DPPH; 0.024 %). The reaction mixture was then incubated for 30 min in the dark conditions and absorbance was read at 515 nm. Trolox (6-hydroxy-2, 5, 7, 8-tetramethylchroman-2-carboxylic acid) was used as a positive control and the results were expressed as mmol Trolox equivalents g^−1^ dry weight (mmol TE g^−1^ DW).

### Determination of stomatal conductance

Stomatal conductance was measured on the third leaf of each plant using a SC-1 porometer (Decagon, Pullman, Washington, DC) from each treatment at each of the different sampling points.

### Quantitative real-time PCR analysis of stress inducible genes

Total RNA was extracted from the control and ANE-treated soybean plants during different stages of the drought treatment using the RNAeasy kit (Qiagen), following the manufacturer’s protocol. The quantity and purity of total RNA was analysed using a NanoDrop 2000 spectrophotometer (Thermo Scientific, USA). The total RNA was stored at −80 °C, and used for qPCR expression analysis. 2.0 μg of RNA was treated with DNase I (Promega, USA) followed by first strand cDNA synthesis using a RevertAid cDNA synthesis kit (Thermo Scientific, USA). Real-time PCR was completed using cDNA by Step One™ Real-Time PCR system (Applied Biosystems). Tubulin was used as an internal control gene. A list of the primers used in this study is presented in Table 1. The specificity of PCR amplification was validated at the end of the PCR cycles, by melt-curve analysis. Each reaction was replicated three times and the relative-fold expression was determined using $2^{-\Delta \Delta C_{t}}$ method as described by Livak and Schmittgen (2001).

**Table 1.** List of the primers used in gene expression analysis.

S.no.	Genes	Primers	Function
1.	Fibrillin 1a	**FiB F:** 5′-TTAGATGCTCGTGCGAATGG-3′	Involved in photoprotection against photoinhibition
		**FiB R:** 5′-CGCTATACTTGGACGAACCTTG-3′	
2.	GmDREB1B	**GmDREB1B F:** 5′-GTAAAGATTGTTCGTATGGGACAAG-3′	Drougt tolerance by regulating expression of *GmPLY21*
		**GmDREB1B R:** 5′-ACACCTAAAATGAGCAACCGTACTA-3′	
3.	GmTP55 (Antiquitin)	**GmTP55 F:** 5′-CGAAAAGGGAGAGGAGGACTTC-3′	Aldehyde dehydrogenase gene involved in drought tolerance
		**GmTP55 R:** 5′-TCTGGGTCACCGAAAGGCAA-3′	
4.	GmBIP	**GmBIPD F:** 5′-ATCTGGAGGAGCCCCAGGCGGTGG-3′	BiP overexpression confers resistance to drought
		**GmBIPD R:** 5′-CTTGAAGAAGCTTCGTCGTAAAACTAAG-3′	
5.	GmGST	**GmGST F:** 5′-CGGTTCTCATCCACAATGGCAAAC-3′	Drought tolerance; Glutathione S transferase
		**GmGST R:** 5′-CAGCCCAGAATCTAGCCTGAGC-3′	
6.	GmRD22	**GmRD22 F:** 5′-AATGCCGAAAGCCATTACAG-3′	Drought tolerance
		**GmRD22 R:** 5′-GCTTTGTTTTCCCTGCGTTA-3′	
7.	GmNFYA3	**GmNFYA3 F:** 5′-ACCAGAATTGCATTACCAGTTGA-3′	Induced by ABA and drought; is a positive regulator of drought tolerance
		**GmNFYA3 R:** 5′-GGTGCCGAGACTCATGAAGATAT-3′	
8.	GmPIP1b	**GmPIP1b F:** 5′-TCATGGGTTTCAAAAAGGAGA-3′	Aquaporins
		**GmPIP1b R:** 5′-GCTTGCAATAAAAGCACAAGC-3′	
9.	GmCYP707A1a	**GmCYP707A1a F:** 5′-GGAAGATGATTGATTACAAGGAC-3′	ABA 8′-hydroxylase induced by dehydration
		**GmCYP707A1a R:** 5′-GACACTGGGGTTTTCACCGA-3′	
10.	GmCYP707A3b	**GmCYP707A3b F**: 5′-GGCTAACCTTCTGACTTTCC-3′	ABA 8′-hydroxylase induced by rehydration
		**GmCYP707A3b R**: 5′-CAAGTGTCTGGTTCTGAGGT-3′	
11.	GmERD1	**GmERD1 F:** 5′-CGTCCAGAATTGCTCAACAG-3′	Dehydration induced
		**GmERD1 R:** 5′-TGGGGTTATAGCCTTGTTGG-3′	
12.	GmRD20	**GmRD20 F:** 5′-GTGGCACATGACTGAAGGAA-3′	Involved in dehydration
		**GmRD20 R:** 5′-ATCTTTCCAGCAGCACCTCT-3′	
13.	Tubulin	**Tubulin F:** 5′-ATGTTCCGTGGCAAGATGAG-3′	Housekeeping gene for reference
		**Tubulin R:** 5′-CATTGTTGGGAATCCACTCG-3′	

### Statistical analysis

Results are expressed as mean ± standard deviation (SD). Each experiment was replicated three times and was conducted in triplicate. The data were analysed using ANOVA, with a *P*-value of ≤ 0.05 using the ‘Proc. mixed procedure’, of the SAS Institute, Inc., Software version 9.3 (SAS Institute, Inc., Cary, NC). When significant effects of treatments were found, a multiple means comparison was carried out using Tukey’s analysis, with a 95 % confidence interval and α = 0.05 to differentiate treatment means.

## Results and Discussion

### ANE improves growth of soybean under drought stress

**Figure 1.**
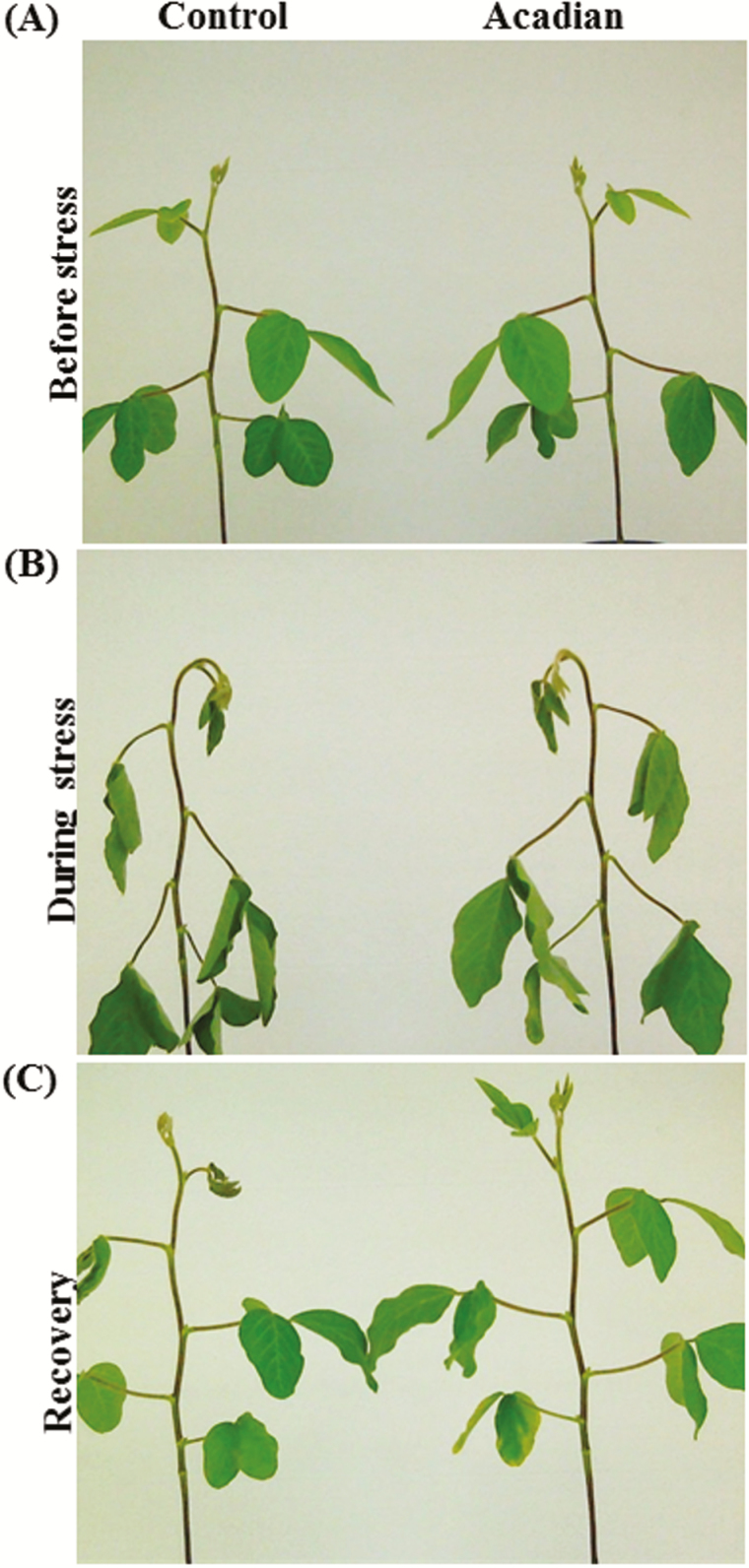
ANE mitigate drought stress in soybean. Twenty-one-day old soybean plants were divided in two sets and treated with 1.0 g L^–1^ 20-8-20 and 1.0 g L^–1^ 20-8-20 plus 7.0 mL L^–1^ ANE, respectively. The plants were subjected to drought stress by stopping irrigation. The plants treated with 1.0 g L^–1^ 20-8-20 fertilizer served as the control. The effect of application of ANE growth of soybean: (a) before onset of drought (22 h after treatment), (b) during drought stress (75 h after treatment) and (c) after recovery (8 h after rewatering plant).

Applications of ANE reduced the degree of wilting of soybean grown under the influences of imposed drought conditions (Fig. 1). The plants treated with ANE had demonstrably better adaptability to recover from drought conditions. ANE treated plants had a 50 % higher water content when exposed to drought conditions, as compared to the control plants (data not shown).

### Effect of application of ANE on stomatal conductance and free-radical scavenging activity in drought-stressed soybean

**Figure 2.**
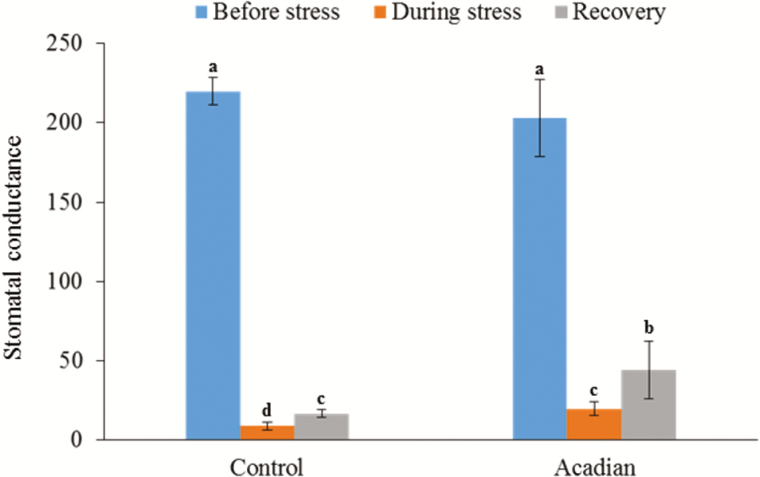
ANE improves stomatal conductance of soybean under drought stress.The graph represents the effect of ANE on stomatal conductance of soybean during different stages of drought stress. The values represented in the graphs were calculated from three independent experiments (*n* = 10). Significantly different values between the treatment and control are represented by different letters.

ANE treatment significantly improved the stomatal conductance of soybean plants under drought conditions (Fig. 2a); the stomatal conductance of ANE treated plants was 46 % greater than that of control plants (Fig. 2a). After rehydration, stomatal conductance of ANE treated plants was 46 % higher than control plants (Fig. 2a). Martynenko *et al.* (2016) showed that ANE from *A. nodosum* improved the drought tolerance of soybean plants by regulation of leaf temperature and turgor. During water scarcity, plants reduce stomatal conductance, and consequently causing an increase in leaf temperature (Grant *et al.* 2006). ANE not only reduced rapid increase in leaf temperature (Martynenko *et al.* 2016), but also improved stomatal conductance, resulting in improved drought tolerance.

**Figure 3.**
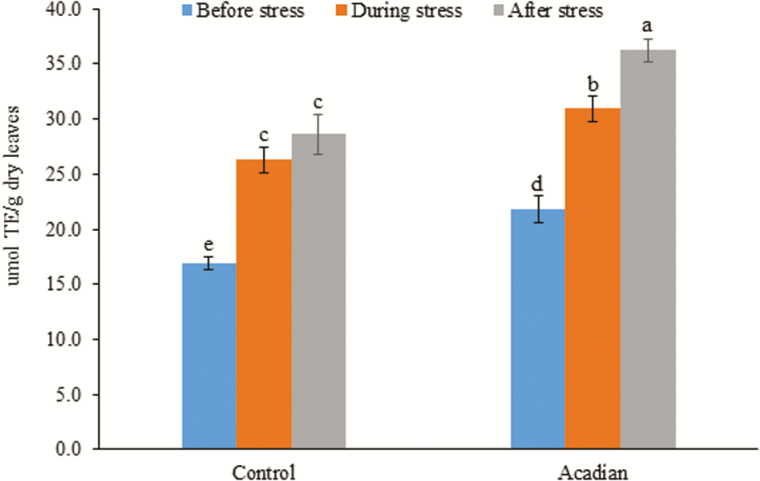
Effect of the application of ANE on free-radical scavenging activity by DPPH assay of soybean during different stages of drought stress. The values represented in the graphs were calculated from three independent experiments (*n* = 10). Significantly different values between the treatment and control are represented by different letters.

The results of radical-scavenging activity by DPPH (1, 1-diphenyl-2-picrylhydrazyl) assay revealed that ANE-treated soybean showed significantly higher inhibition of reactive oxygen species (ROS)-scavenging capacity, as compared to the controls, during severe drought conditions (20 %), and importantly after rehydration (27 %) as well (Fig. 3b), suggesting ANE mitigates drought tolerance in soybean by improving ROS-scavenging capacity of the treated plants (Fig. 3b).

### Drought tolerance of ANE-treated soybean by the regulation of stress-responsive gene expression

There was no significant difference between the control and ANE-treated plants in the expression of any of the genes investigated before stress.

**Figure 4.**
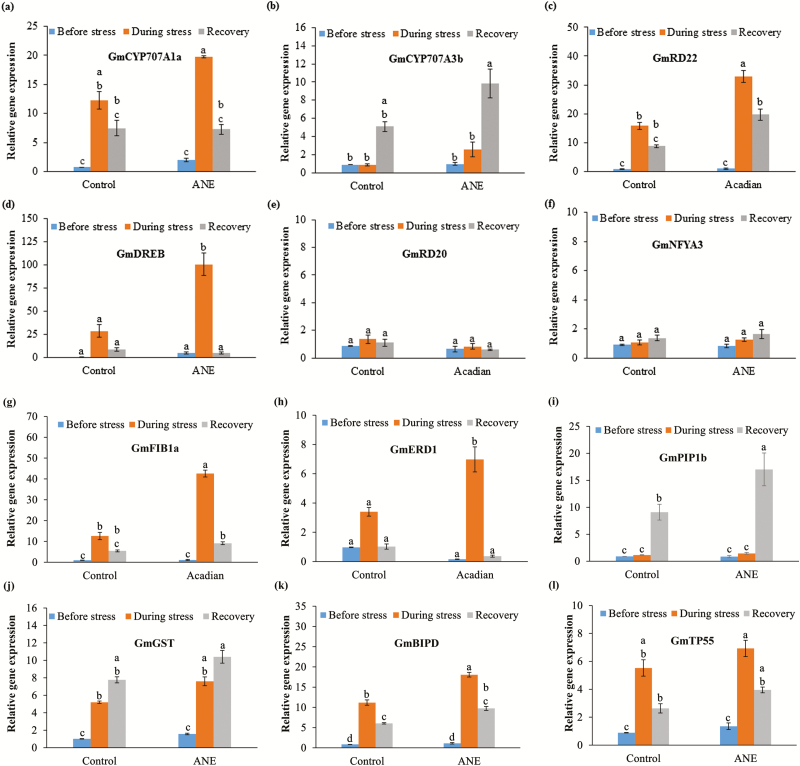
Effect of ANE on the relative fold expression of genes of selected soybean genes during different stages of drought stress by qPCR: (a) *GmCYP707A1a*, (b) *GmCYP707A3b*, (c) GmRD22, (d) *GmRD20*, (e) *GmDREB1B*, (f) GmERD1, (g) *GmNFYA3*, (h) *FIB1a*, (i) *GmPIP1b*, (j) *GmGST*, (k) *GmBIP* and (l) *GmTp55*. The values represented in the graphs were calculated from three independent experiments (*n* = 3). Significantly different values between the treatment and control are represented by different letters.

Applications of ANE improved stomatal conductance by regulating the ABA biosynthesis pathways. ABA plays an important role in plant responses to drought stress (Zheng *et al.* 2012). *GmCYP707A1a* and *GmCYP707A3b*, identified as ABA 8′-hydroxylases, which are known to play a central role in regulating ABA levels during dehydration and rehydration, respectively (Umezawa *et al.* 2006; Zheng *et al.* 2012). Treatment of soybean plants with the 7 mL L^–1^ of ANE induced the expression of *CYP707A1a* by 1.6-fold during dehydration (Fig. 4a), while the expression of *CYP707A3b* was significantly increased by 1.9-fold after drought relief in the treated soybean plants (Fig. 4b). Thus, treatment with ANE modulate the biosynthesis of ABA by regulating the expression of *CYP707A* genes during both dehydration and rehydration. ANE-treated soybean showed a significant increase in the expression of *GmRD22* (2-fold increase) during the applied drought stress (Fig. 4c). *GmDREB1B*, an ABA-dependent DRE-binding transcription factor involved in plant drought tolerance, showed a 3.5-fold greater expression in the drought-stressed ANE-treated plants (Fig. 4d). In contrast, *GmRD20*, an ABA-independent stress responsive gene was not induced by the extract applications (Fig. 4e). This suggested that the ANE treatment-induced, ABA-dependent regulation of stomata (via regulating expression of *GmRD22*) helped plants to withstand those drought conditions. In addition to this, the greater expression of *GmRD22* is able to induce the expression of cell wall peroxidases and improve cell wall integrity under the stress conditions (Wang *et al.* 2012). The expression of *GmNFYA3*, which encodes for nuclear factor Y subunit of the NF-Y complex (Ni *et al.* 2013), was found to remain unaltered in ANE-treated plants (Fig. 4f).

It is postulated that in plants treated with 7 mL L^–1^ of ANE, ABA induced fibrillin accumulation and the ABA-response regulators (ABI1, ABI2) regulated the fibrillin expression. Fibrillins are lipid binding proteins of the plastid involved in abiotic stress tolerance (Yang *et al.* 2006). ANE extract at the rate applied, significantly induced the expression of *FIB1a* during drought, as compared to the control plants (Fig. 4g). The higher expression of *FIB1a* in Arabidopsis was shown to enhance photosystem II (PSII) photo-tolerance, while reducing *FIB1a* expression which decreases PSII photo-tolerance (Mutava *et al.* 2015). ANE-treated plants showed relatively higher transcripts of *FIB1a*, resulting in increased photo-tolerance and reduced stress-induced photo-inhibition of PSII. The expression of *FIB1a* is regulated by ABA, and is involved in ABA-mediated photo-protection during stress (Yang *et al.* 2006). Thus, taking all of these together we conclude that treatment with ANE improved the drought tolerance of soybean plants by ABA-induced photo-protection, by regulating *FIB1a*. In contrast to other genes evaluated in this work, *GmERD1*, which belongs to the ABA-independent pathway, was also induced 2-fold in the ANE-treated soybeans under the drought conditions tested (as compared to the control) (Fig. 4h).

Aquaporins are family of small integral membrane proteins which acts as an osmo-sensor in plant membranes involved in the control of inter-cellular water movement between cells during drought stress (Stolf-Moreira *et al.* 2010). Application of ANE extract (at the rates tested) helped soybean plants in recovering from drought stress by increasing the expression of *GmPIP1b* by 1.8-fold during the recovery phase (as compared to control; Fig. 4i). Those plants treated with ANE showed an increased transcript abundance of *GmPIP1b* during drought stress and yet maintained internal water movement in the plants during the water stress condition. Drought stress in plants leads to the generation of ROS at the chloroplast PSI and PSII and the mitochondrial complex I and III of the electron transport chain. Glutathione S transferase (GST) functions to remove ROS during stress. Under the conditions of this study, the expression of GST increased in the ANE treatments, as compared to controls, but that change was not significant (Fig. 4j). *GmGST* protect cell from oxidative damage by quenching reactive molecules with the addition of glutathione (McGonigle *et al.* 2000). *GmBIP*, a molecular chaperone that increase drought tolerance in soybean by delaying leaf senescence (Valente *et al.* 2009), was induced in ANE-treated plants (as compared to control), during drought stress, as well as the recovery stages (Fig. 4k). Thus, as demonstrated here, application of ANE mediates the drought response in soybean by regulating expression of *GmBIP* and *GmGST,* and protected treated plants by delaying drought-induced senescence and reduced any oxidative damage to tissues. *GmTP55*, an antiquitin-like soybean protein was expressed significantly higher in ANE-treated plants during drought stress, however, the increase was not significantly different during the recovery stage (Fig. 4i). Therefore, it is possible that ANE-induced *GmTP55* could be involved in the adaptive response by reducing lipid peroxidation-derived, reactive aldehydes, under oxidative stress (Rodrigues *et al.* 2006).Thus, under the rate of application and drought conditions presented in this study, ANE was able to mitigate the drought stress by regulating the expression of genes involved in ABA biosynthesis and ROS detoxification.

The fact that there was no significant difference in gene expression between treated and control plants before exposure to drought stress suggests that ANE only activates the drought resistance mechanisms of the plant when the plants are under stress. With the exception of genes such as *GmPIP1b*, *GmGST* and *GmCYP707A3b* which showed higher expression in both control and treated plants, the level of expression in the ANE-treated plants in the recovery time point was already decreasing compared to the period during stress, again indicating that the response is only present while the plants are under stress. This is important, as drought resistance mechanisms such as stomatal closure can lead to reduced productivity if extended beyond the period of stress (Neumann 2008).

Of the genes investigated, *GmRD20* and *GmNFYA3* showed little response to the drought stress in either control or treated plants. This suggests that the mode of action of ANE works by amplifying the plants’ natural response to drought stress in terms of increased gene expression. Thus, our physiological and gene expression study revealed that treatment with 7 mL L^–1^ ANE resulted in the successful adaptation and survival of treated soybean plants under the conditions tested (as compared to control).

## Conclusions

In conclusion, the use of ANE in agriculture presents a promising approach to improve plant growth and impart drought tolerance. In addition, more comprehensive and systematic studies of functionality at the molecular level, in model plant systems such as Arabidopsis, are required to further decipher modes of action during drought tolerance. These approaches may lead to the discovery of plant-target molecules that interact with the bioactive components of *A. nodosum,* and hence reveal novel regulatory interactions involved in drought tolerance.

## Sources of Funding

The work reported in this article was funded in part by Industry Research Assistance Program of the National Research Council of Canada.

## Contributions by the Authors

B.P., W.N. and A.T. conceived and conceptualized the idea. P.S.S., K.S., E.N., W.N., A.T. and B.P. designed experiment. P.S.S., K.S. and E.N. performed experiments and analyzed data. P.S.S., K.S., A.T. and B.P. interpreted the data and prepared the manuscript.

## Conflict of Interest

None declared.
